# Physicochemical properties of bone marrow mesenchymal stem cells encapsulated in microcapsules combined with calcium phosphate cement and their ectopic bone formation

**DOI:** 10.3389/fbioe.2022.1005954

**Published:** 2022-10-06

**Authors:** Yafei Yuan, Lipei Shen, Tiankun Liu, Lin He, Dan Meng, Qingsong Jiang

**Affiliations:** ^1^ Beijing Stomatological Hospital, School of Stomatology, Capital Medical University, Beijing, China; ^2^ Biomanufacturing Center, Department of Mechanical Engineering, Tsinghua University, Beijing, China

**Keywords:** microcapsule, calcium phosphate cement, bone marrow mesenchymal stem cells, scaffold, tissue engineering

## Abstract

Calcium phosphate bone cement (CPC) serves as an excellent scaffold material for bone tissue engineering owing to its good biocompatibility, injectability, self-setting property and three-dimensional porous structure. However, its clinical use is limited due to the cytotoxic effect of its setting reaction on cells and difficulties in degradation into bone. In this study, bone marrow mesenchymal stem cells (BMSCs) were encapsulated in alginate chitosan alginate (ACA) microcapsules and compounded with calcium phosphate bone cement. Changes in the compressive strength, porosity, injectability and collapsibility of CPC at different volume ratios of microcapsules were evaluated. At a 40% volume ratio of microcapsules, the composite scaffold displayed high porosity and injectability with good collapsibility and compressive strength. Cell live/dead double staining, Cell Counting Kit-8 (CCK-8) assays and scanning electron microscopy were used to detect the viability, proliferation and adhesion of cells after cell microcapsules were combined with CPC. The results revealed that cells protected by microcapsules proliferated and adhered better than those that were directly combined with CPC paste, and cell microcapsules could effectively form macropores in scaffold material. The composite was subsequently implanted subcutaneously on the backs of nude mice, and ectopic osteogenesis of the scaffold was detected *via* haematoxylin-eosin (H&E), Masson’s trichrome and Goldner’s trichrome staining. CPC clearly displayed better new bone formation function and degradability after addition of pure microcapsules and cell microcapsules. Furthermore, the cell microcapsule treatment group showed greater osteogenesis than the pure microcapsule group. Collectively, these results indicate that BMSCs encapsulated in ACA microcapsules combined with CPC composite scaffolds have good application prospects as bone tissue engineering materials.

## Introduction

Calcium phosphate cement (CPC) has significant similarities with hydroxyapatite, the main component of human bone. Soluble calcium and inorganic phosphate generated by the decomposition of calcium phosphate crystals can be effectively used by cells to form new bone (Wang et al., 2014a; Thrivikraman et al., 2017) and are widely applied as synthetic bone graft materials. The injectability and self-setting ability of CPC facilitates injection into irregular bone defect cavities using minimally invasive surgery and solidification *in situ* to fit the shape of the bone defect, which reduces the shaping time of the graft material and trauma of the operation (Alves et al., 2008; Low et al., 2010; Liu et al., 2016). However, poor degradability and osteoinductivity properties greatly limit its applications. Over the years, researchers have attempted to improve the biocompatibility, bone induction and porosity of CPC by adding bioactive factors, metal and nonmetal ions, organic compounds, drugs or stem cells (Wang et al., 2014b). However, this issue has not been resolved, so this material is not commonly used in oral treatment.

BMSCs are widely used in stem cell therapy owing to their self-renewal ability, rapid proliferation *in vitro*, rich separation sources, immune regulatory ability, and multidirectional and high osteogenic differentiation (Chu et al., 2020; Mohanty et al., 2020; Purwaningrum et al., 2021). In bone tissue engineering applications, bone marrow mesenchymal stem cells are often used to generate composite biomaterials with scaffolds to improve osteogenic properties and promote new bone formation (Liu et al., 2010; Wang et al., 2013; Chen et al., 2014; Lee et al., 2019; Arthur and Gronthos, 2020; Oryan et al., 2020). However, CPC paste exerts toxic effects during the setting reaction, which is not conducive to the growth of adhesion cells on its surface (Zhao et al., 2010). Simon et al. (2004) reported that during the process of solidification, cell survival was enhanced in cases where cells were not in direct contact with CPC.

Therefore, to solve the above problems, this study used microencapsulation (ACA)-encapsulated BMSCs and mixed them with CPC to form an injectable composite. On the one hand, encapsulating BMSCs in microcapsules can isolate cells from CPCs, prevent the toxicity of CPC paste, and improve cell viability, thereby further enhancing the osteogenic properties of composites. Due to the unique microenvironment and immune isolation, microencapsulation technology shows potential for tissue engineering and regenerative medicine (Xia et al., 2012; Wang et al., 2014c). Alginate and chitosan have been used to generate multilayered hollow microcapsules due to their good biocompatibility, low cost and similar structure to the extracellular matrix (Ribeiro et al., 2018). ACA microcapsules appear to be the perfect choice to provide cells for tissue engineering. Zhang et al. (2017) used AC microcapsules encapsulating HUVEC-CS cells to construct vascularized tissues. Long et al. (2017) comicroencapsulated BMSCs and mouse pancreatic β cells for the treatment of diabetic mice. However, there are few related studies on the application of ACA microcapsules in the oral cavity. On the other hand, the use of ACA to increase the porosity of CPC results in a three-dimensional cell-carrying scaffold with interconnected micropores and macropores and improves its osteogenic properties. Previous studies have found that in addition to the micropores (<50 μm) of CPC itself, which can increase the area of protein adsorption and cell attachment, the interconnected macropores (>100 μm) formed by microcapsules are important for bone ingrowth, blood vessel crawling, cell migration, and nutrition delivery (Grosfeld et al., 2020). Therefore, this study will utilize a simple and efficient electrospraying method for the preparation of BMSC-loaded microcapsules mixed with CPC to form an injectable composite that can be applied in the oral cavity.

## Materials and methods

### Preparation of alginate-chitosan-alginate-encapsulated bone marrow mesenchymal stem cells and alginate-chitosan-alginate/calcium phosphate cement composite scaffolds

Alginate-chitosan-alginate (ACA) microcapsules were prepared by an electrospraying method. Sodium alginate (1.5%; A0682, Sigma) was injected through a 10 ml syringe, which was fixed on a syringe pump (LSP02-1B, Longer Pump, China) and connected to the positive pole of a DC electric field (BGG, BME I Co., Ltd. China). With the basic parameters of a voltage of 5.0 kV, the syringe pump was started at a speed of 15 ml/h, a receiving distance of 2 cm, and a pinhole diameter of 0.21 mm. Then, 1.5% (w/v) sodium alginate solution was dropped evenly into 1.1% (w/v) calcium chloride solution (Shanghai Test, China) to obtain calcium alginate microspheres. The microspheres formed were successively reacted with 0.6% (w/v) chitosan (C8320, Solarbio, China; molecular weight: 100,000, deacetylation>90%, pH 6.3) for 15 min and 0.05% (w/v) sodium alginate solution for 5 min, washed with normal saline (NS), and liquefied with 55 mmol/L sodium citrate solution (pH 5.6, Shanghai Test) for 10 min to obtain ACA microcapsules. When 10^6^/ml bone marrow mesenchymal stem cells (BMSCs) were added to 1.5% (w/v) sodium alginate and the other steps were the same, cell microcapsules were prepared.

CPC powder was composed of α-TCP [α-Ca_3_(PO_4_)_2_], calcium dihydrogen phosphate monohydrate [Ca(H_2_PO_4_)_2_H_2_O] and calcium carbonate (CaCO_3_) at a 10:3.5:1.5 M ratio, and the solidifying liquid used was sodium dihydrogen phosphate (NaH_2_PO_4_)/sodium hydrogen phosphate (Na_2_HPO_4_) solution at an equal molar ratio. The powder/liquid ratio was 1 g:1 ml. ACA microcapsules were mixed with CPC at proportions of 0%, 20%, 30%, 40%, and 50% (v/v) with solidifying liquid. The final CPC samples were labelled CPC0%, CPC20%, CPC30%, CPC40% and CPC50% according to the ACA microcapsule proportion.

### Injectability test

A 5 ml syringe containing different ratios of microcapsules mixed with CPC was placed in a universal testing machine (Autograph AG-X plus; Shimadzu, Japan) and fixed vertically, and the biomaterial passed through at a rate of 10 mm/min until the pushing force reached 100 N. The total mass of the material was recorded as M1, and the mass of the remaining material after pushing was recorded as M2. Injectability was calculated as (M1—M2)/M1*100%.

### Compressive strength test

For determination of the compressive strength of the composite scaffold, CPC0%, CPC20%, CPC30%, CPC40% and CPC50% were placed in moulds with a diameter of 6 mm and height of 10 mm to generate standard specimens (*n* = 4), which were loaded in a universal testing machine to determine compression resistance at a speed of 1 mm/min.

### Anti-washout testing

Each group of composite biomaterials was loaded into a 5 ml syringe, injected in NS, and incubated at 37°C and >90% humidity. Images were obtained at 0 s and 24 h to examine collapse of the biomaterials.

### Evaluation of porosity

The Archimedes drainage method was employed to evaluate the porosity of all specimens. The dry weight of each sample was determined (recorded as M0). Samples were placed in a clean beaker, submerged in water and heated to the boiling point for 2 h until the water completely penetrated each sample. After termination of heating, the samples were cooled to room temperature, and weights were obtained (recorded as M1). Following removal of each sample, moisture on the surface was wiped off, and the weight was recorded (M2). Porosity was calculated as (M2-M0)/M1. The pore distribution of the composite scaffold was examined using FE-SEM (Hitachi S-4800; Hitachi, Tokyo, Japan).

### Cell live/dead double staining

Cells of the same density (10^6^ cells/ml) were encapsulated into ACA microcapsules and composited with CPC or directly composited with CPC. At 24 h, the composite scaffolds were carefully broken, and the cells were harvested by a cell strainer. The cells in the ACA-C (ACA microcapsules purely encapsulating BMSCs), CPC + ACA-C (composite of cell microcapsules and CPC paste) and CPC + C (cells and CPC paste composite) groups were plated and live/dead stained (CA1630, Solarbio, China) at 24 h. The experimental results were observed and photographed under a laser scanning confocal microscope (ECLIPSE-Ti, Nikon, Japan). The percentage of live cells was P_Live_ = N_Live_/(N_Live_ + N_Dead_). Experimental results were statistically analysed.

### Cell attachment assay

Electron microscopy was used to examine the adhesion of bone marrow mesenchymal stem cells on the CPC surface. The cells were divided into the CPC paste (BMSCs seeded on CPC paste), CPC disc (BMSCs seeded on CPC disc), and CPC + ACA-C groups (BMSCs encapsulated in ACA microcapsules seeded in CPC paste). Each group of composites was suspended in culture medium, incubated at 37°C and 5% CO2 for 24 h, and subsequently fixed in 2.5% glutaraldehyde. After dehydration, drying and gold spraying, the adhesion of cells from each group on the surface of bone cement was examined under an electron microscope.

### Cell proliferation analysis

The CCK-8 assay was employed to assess the proliferation of cells. The cells were divided into the control (adherent culture of BMSCs), ACA-C (ACA microcapsules purely encapsulating BMSCs), CPC + ACA-C (composite of cell microcapsules and CPC paste), and CPC + C groups (cells and CPC paste composite). All four groups of cells were plated, and CCK-8 detection was performed on Days 1, 3, and 5 (*n* = 5). Experimental results were plotted and statistically analysed.

### Ectopic bone formation analysis

All surgeries were performed in accordance with a protocol approved by the Animal Welfare Committee of the Beijing Stomatological Hospital, Capital Medical University. Twenty-one 6-week-old male nude mice were used as hosts. Experimental mice were divided into four groups (*n* = 5). Mice were anaesthetized intraperitoneally with 5% chloral hydrate (10 ml/kg body weight). CPC, CPC + C, CPC + ACA and CPC + ACA-C biomaterials were implanted subcutaneously into the backs of nude mice, and the back of each nude mouse was replanted at three sites. Experimental animals were sacrificed after 4, 8 and 12 weeks for harvesting of the specimens. The obtained specimens were fixed in 10% neutral buffered formaldehyde for 2 days, decalcified, dehydrated, embedded, sliced and subjected to H&E, Masson’s trichrome and Goldner’s trichrome staining. Images of stained sections were obtained under a microscope (BX61, Olympus, Japan).

### Statistical analysis

All results are expressed as the mean ± standard deviation (SD). The experimental data of each group showed a normal distribution and homogeneity of variance. One-way analysis of variance (ANOVA) was performed to determine significant effects of the variables. *p* values < 0.05 were considered statistically significant.

## Results

### Alginate-chitosan-alginate microcapsules

**FIGURE 1:**
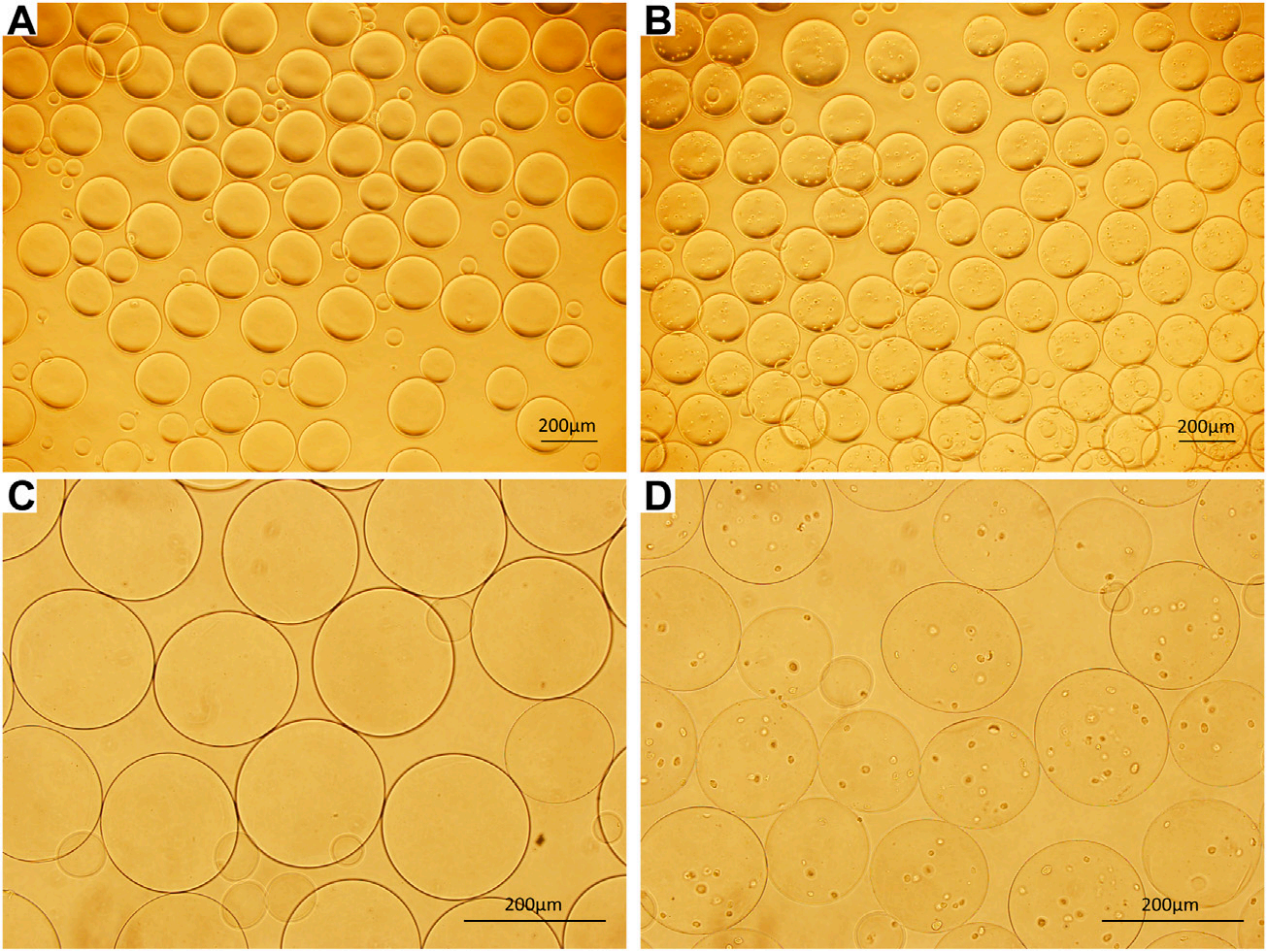
**(A)** ACA microcapsules (40×). **(B)** Cell microcapsules (40×). **(C)** ACA microcapsules (100×). **(D)** Cell microcapsules (100×), bar = 200 μm.

Microcapsules with uniform sizes and diameters of approximately 200–400 µm were prepared by electrospraying (Figure 1).

### Injectability of the alginate-chitosan-alginate/calcium phosphate cement scaffold

**FIGURE 2:**
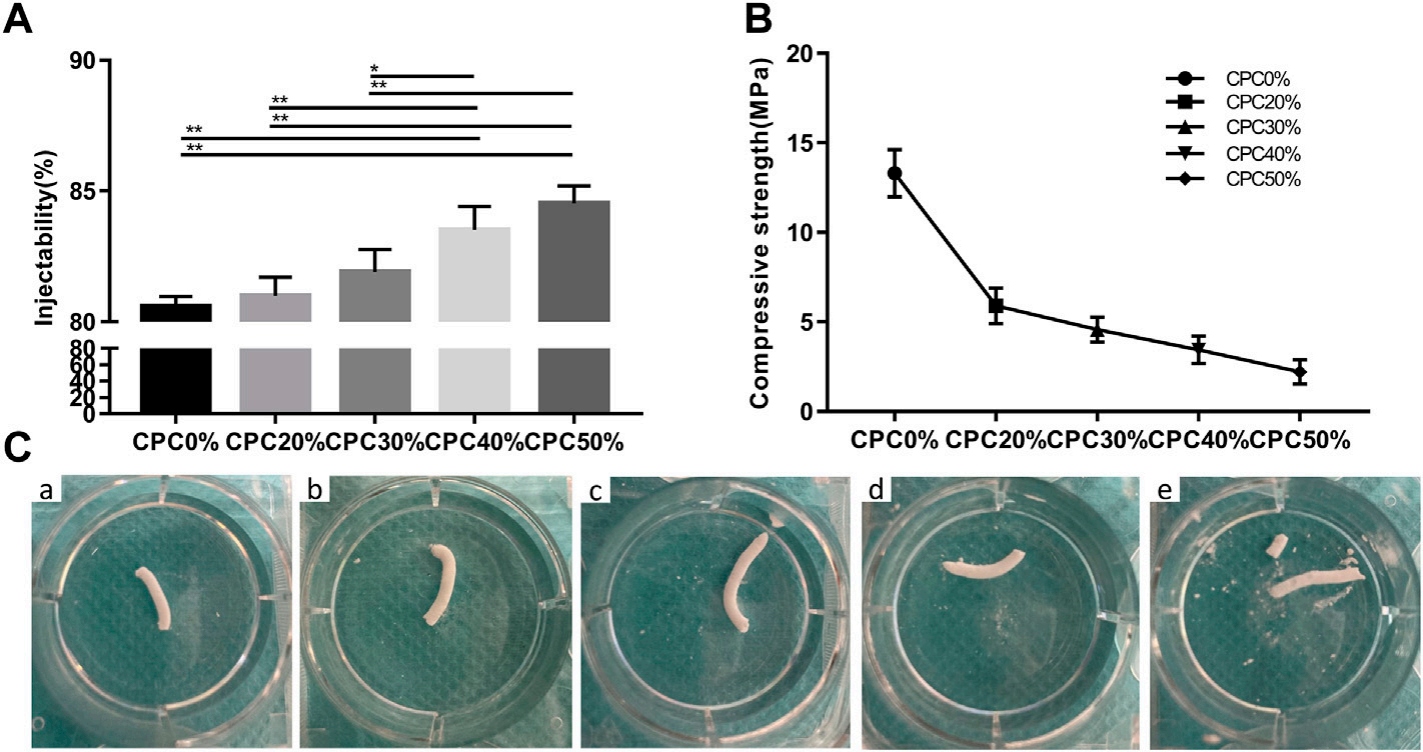
**(A)** Injectability of composite scaffolds with different microcapsule volume ratios. **(B)** Compressive strengths of composite scaffolds with different microcapsule volume ratios. **(C)** Images of composite scaffolds with different microcapsule volume ratios at 24 h after injection of normal saline. **(A)** CPC0%, **(B)** CPC20%, **(C)** CPC30%, **(D)** CPC40%, **(E)** CPC50%. (*: *p* < 0.05, **: *p* < 0.01).

The injectability of composite scaffolds with varying microcapsule volumes is presented in Figure 2A. Injectability experiments revealed an increase in the injectability coefficient with increasing microcapsule proportions. Notably, only the injectability coefficients of CPC40% and CPC50% were significantly different from those of CPC0%. However, the differences between the CPC40% and CPC50% groups were not significant.

### Compressive strength of the alginate-chitosan-alginate/calcium phosphate cement scaffold

Figure 2B shows the compressive strength of each group of composite scaffolds. The compressive strength of bone cement decreased gradually with the increase in microcapsule volume ratio, but the strength of each group still met that of jaw cancellous bone (2–20 MPa), which can be effectively used for bone transplantation in nonload-bearing areas of the jaw.

### Anti-washout ability of the alginate-chitosan-alginate/calcium phosphate cement scaffold

The images of each composite scaffold group at 24 h after injection of normal saline are depicted in Figure 2C. At a microcapsule volume of ≤ 40%, the composite scaffold continued to maintain a good shape with effective anti-washout ability at 24 h. However, at a 50% microcapsule volume ratio, the composite scaffold partially collapsed.

### Porosity of the alginate-chitosan-alginate/calcium phosphate cement scaffold

**FIGURE 3:**
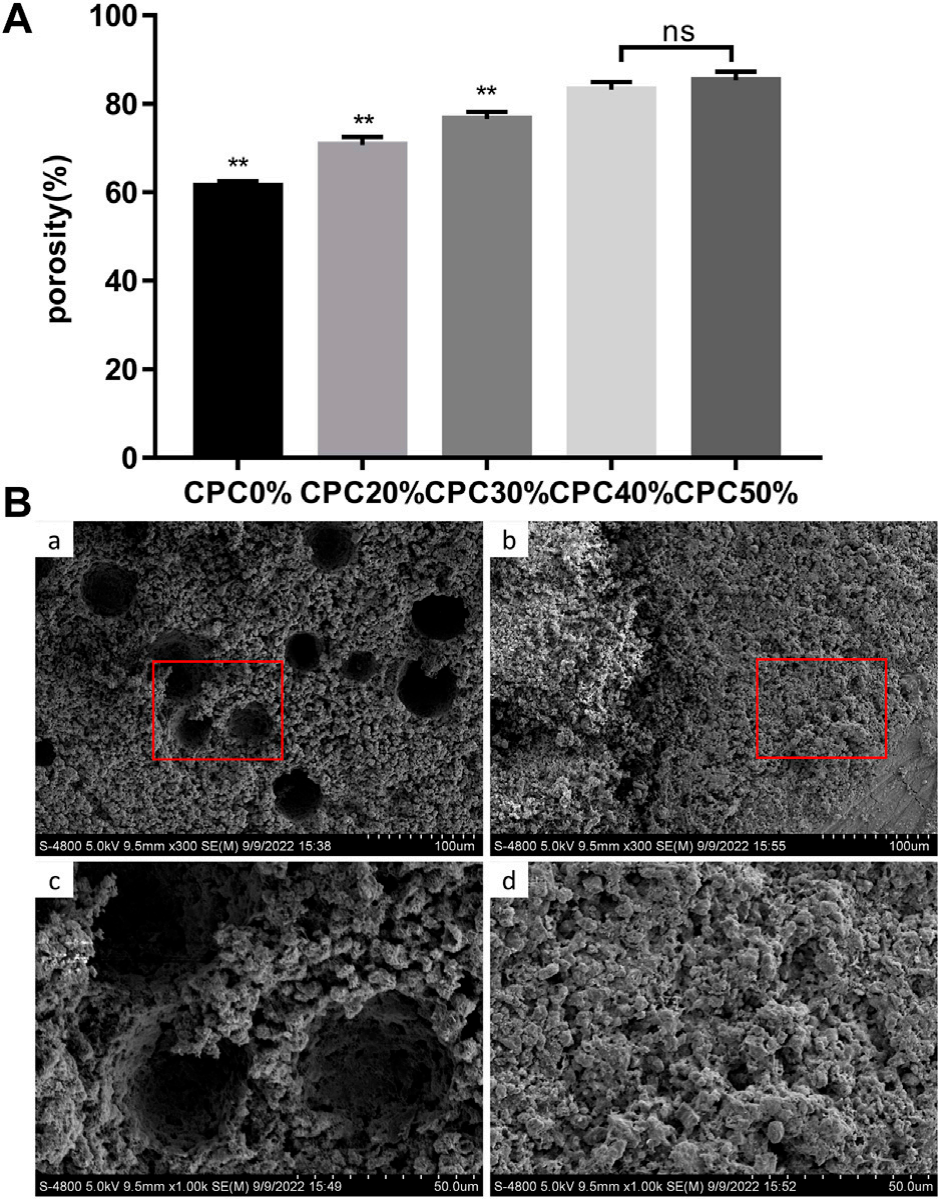
**(A)** Porosity of composite scaffolds with different microcapsule volume ratios measured with the Archimedes drainage method. **(B)** Pore distribution of composite scaffolds observed *via* electron microscopy: **(A)** and **(C)** CPC + ACA, **(B)** and **(D)** CPC. (*: *p* < 0.05, **: *p* < 0.01, ns: *p* > 0.05).

The porosity values of each group of composite scaffolds measured with the Archimedes drainage method are presented in Figure 3A. The porosity clearly increased with the microcapsule volume ratio. Notably, there was a significant difference between each group except the CPC40% and CPC50% groups. In electron microscopy analysis of the cross-sections of composite biomaterials (Figure 3B), CPC displayed 23.706 ± 20.323 µm microporous structures, while the cross-section of CPC combined with microcapsules revealed macropores with diameters of 305.701 ± 103.299 µm in addition to micropores. At the bottom of the macropores, the micropores could also be seen communicating with the surrounding region.

### Cell viability

**FIGURE 4:**
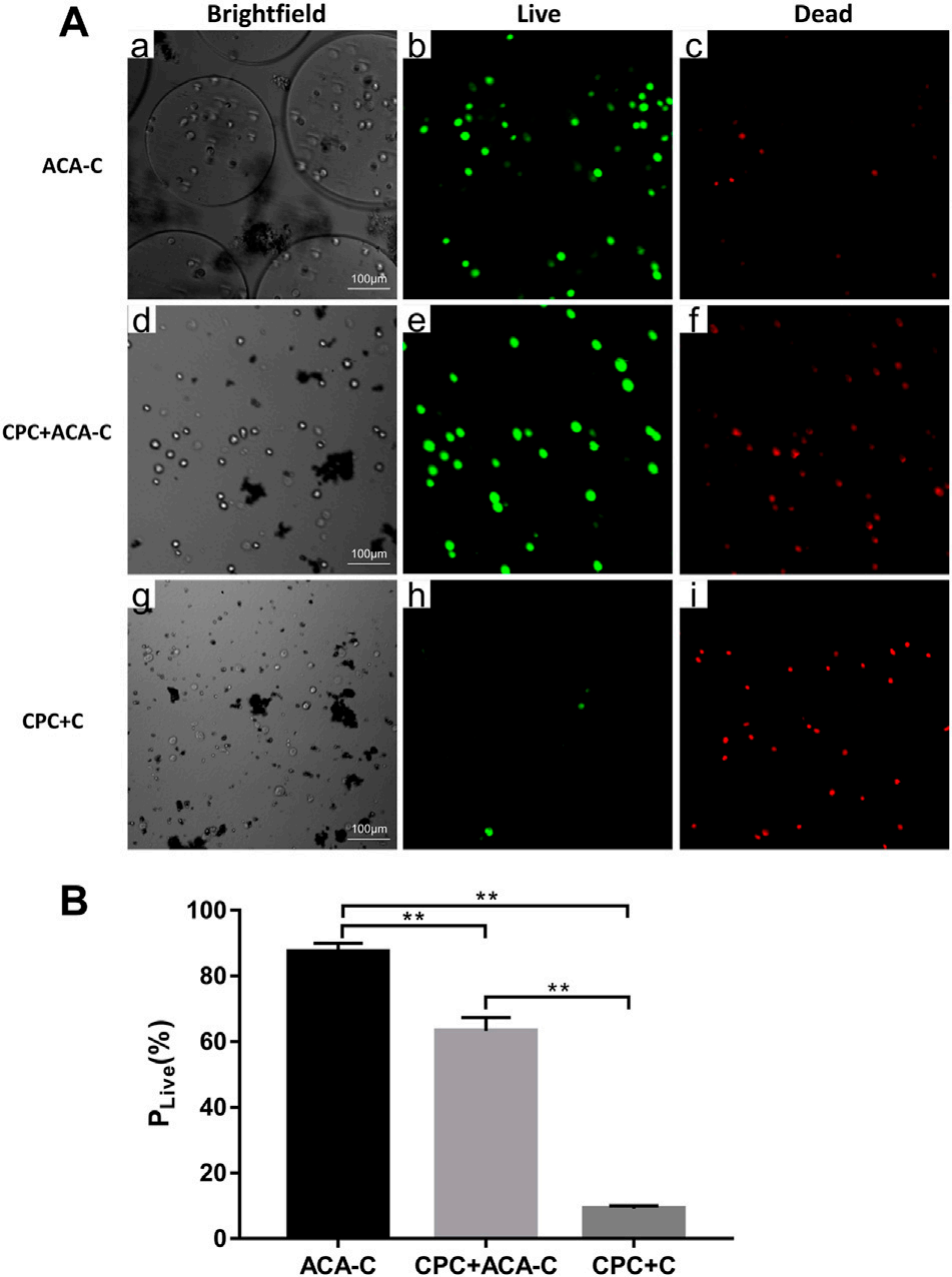
**(A)** Live/dead-stained ACA-C, CPC + ACA-C and CPC + C cells observed *via* laser scanning confocal microscopy. The live cells were green, and the dead cells were red, bar = 100 μm. **(B)** Percentage of live cells in the ACA-C, CPC + ACA-C and CPC + C groups (**: *p <* 0.01).

The live/dead staining results are shown in Figure 4. Under a laser scanning confocal microscope, the live cells were green, and the dead cells were red. In the ACA-C group, most of the cells survived, and the percentage of live cells was 87.455% ± 2.511%. In the CPC + ACA-C group, due to the setting reaction of CPC, some cells died, and the percentage of live cells was 63.26% ± 4.15%. However, in the CPC + C group, a large number of cells died, and the percentage of live cells was 9.099% ± 0.971%. There was a statistically significant difference between each group (**: *p* < 0.01).

### Cell adhesion

**FIGURE 5:**
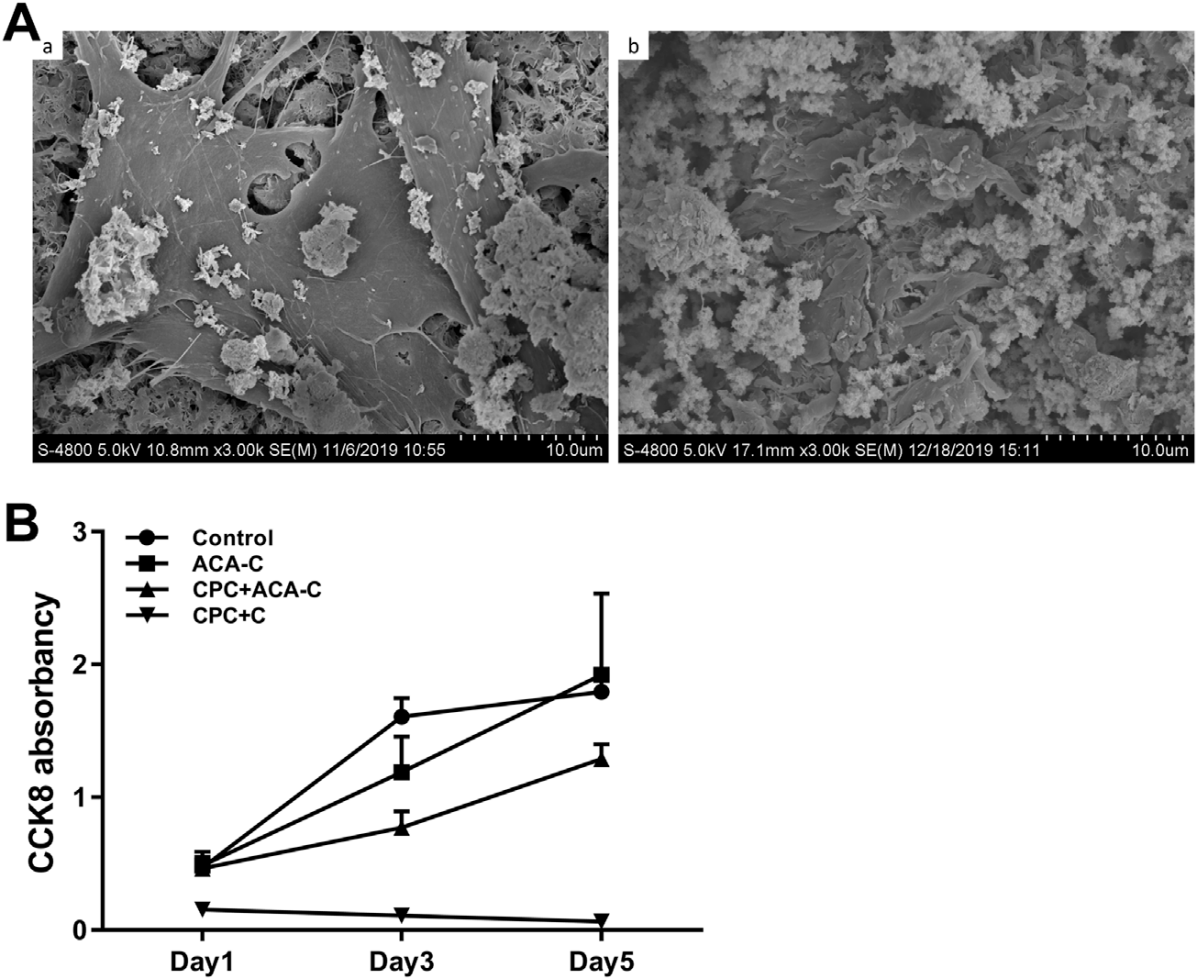
**(A)** Adhesion of BMSCs on the CPC surface observed *via* electron microscopy. **(A)** BMSCs seeded on CPC discs, **(B)** BMSCs encapsulated in ACA microcapsules seeded on CPC paste. **(B)** CCK-8 results of the control, ACA-C, CPC + ACA and CPC + C groups at 1, 3, and 5 days.

The adhesion of cells on the CPC surface was examined *via* electron microscopy after 24 h. Cells did not adhere to the CPC surface upon direct seeding onto CPC paste. In the CPC disc group, BMSCs could be tiled on the surface of CPC. Obvious cell pseudopodia and bulges were detected under a high-power microscope (Figure 5A). Upon encapsulation of cells in microcapsules in combination with CPC paste, cells adhered to the surface of CPC, but the morphology was more three-dimensional, and cell pseudopodia were not obvious (Figure 5(Ab)).

### Cell proliferation

Data from the CCK-8 assay are presented in Figure 5B. We observed stable proliferation in the control and ACA-C groups. Cells in the CPC + ACA-C group showed proliferation in the prophase and proliferated slowly in the anaphase, while those in the CPC + C group showed large-scale cell death.

### Histopathology

**FIGURE 6:**
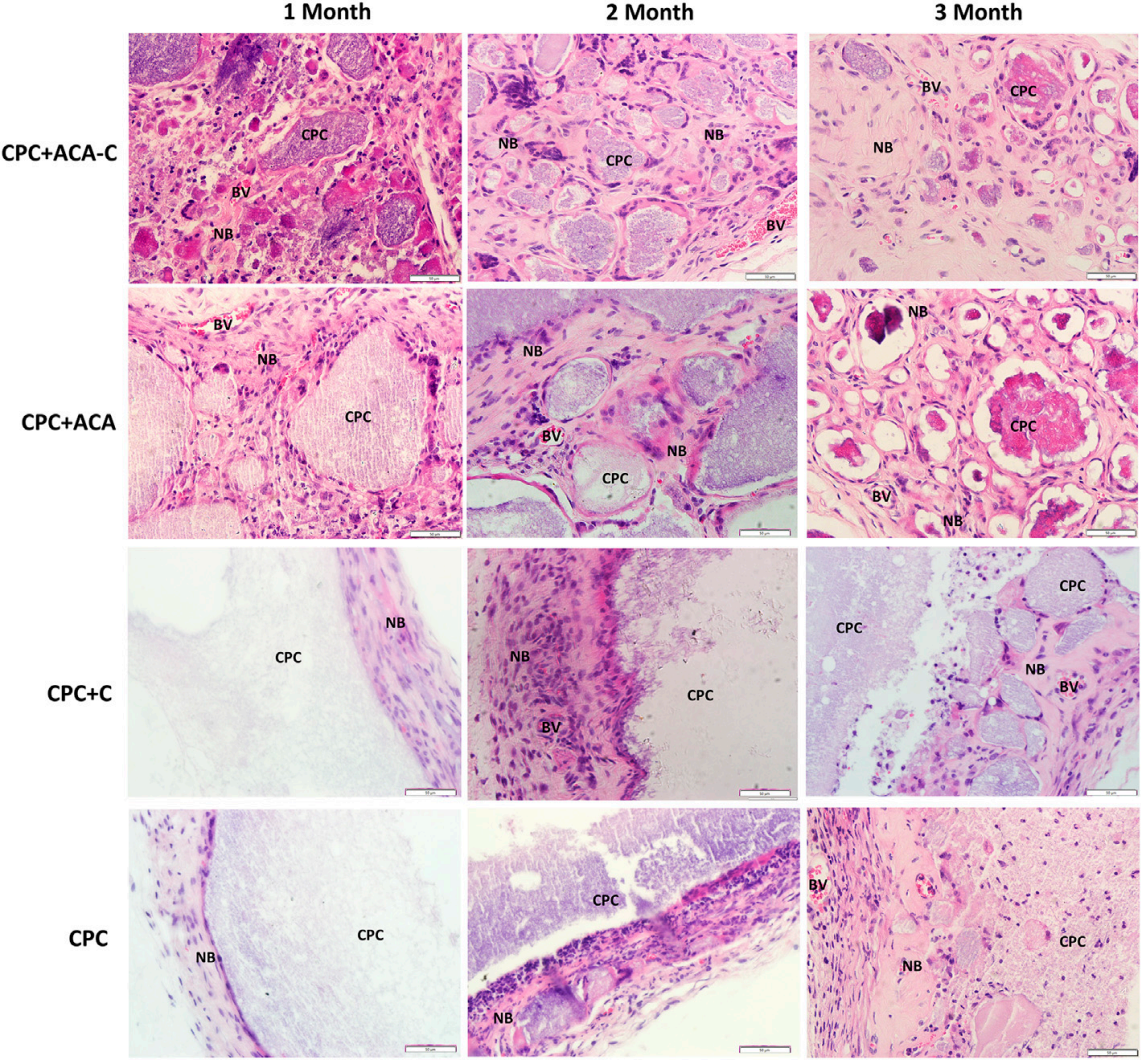
H&E-stained tissue sections of the CPC + ACA-C, CPC + ACA, CPC + C and CPC groups after 1, 2 and 3 months of implantation (NB: newly formed bone; CPC: implanted samples; BV: blood vessel; bar = 50 μm).

**FIGURE 7:**
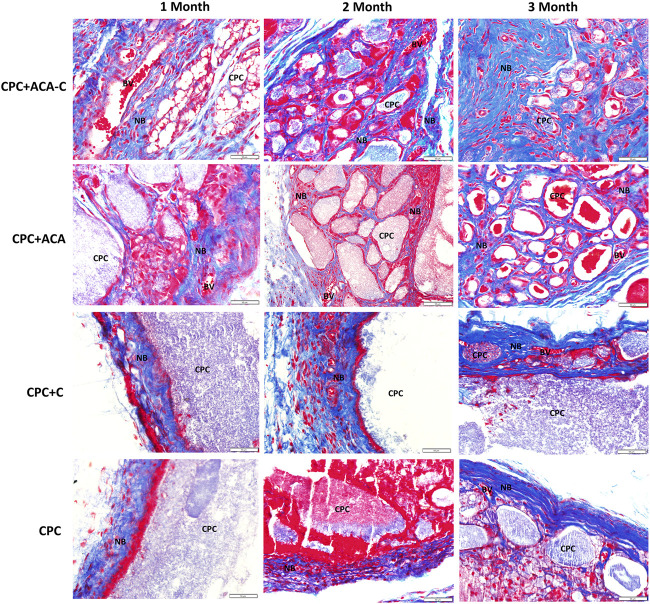
Masson’s trichrome-stained tissue sections of the CPC + ACA-C, CPC + ACA, CPC + C and CPC groups after 1, 2 and 3 months of implantation (NB: newly formed bone; CPC: implanted samples; BV: blood vessel; bar = 50 μm).

The H&E and Masson’s trichrome staining results of subcutaneous replantation in nude mice are shown in Figures 6, 7. At 1 month, only a small amount of new bone-like tissue was observed in each group. In the CPC and CPC + C groups, cells mainly surrounded the periphery of the scaffold, with some protruding from the periphery into the scaffold. In the CPC + ACA and CPC + ACA-C groups, a large proportion of cells infiltrated and degraded the scaffold. Staining at 2 months revealed immature newly formed bone tissue deposition and segmentation around the nondegraded scaffold in the CPC + ACA and CPC + ACA-C groups. In the CPC and CPC + C groups, immature newly formed bone tissue was observed only at the periphery of the scaffold, with infiltration of some peripheral cells. Data from staining at 3 months showed an increase in new bone-like tissue in the CPC + ACA and CPC + ACA-C groups, along with degradation of the scaffold into smaller fragments. In the CPC and CPC + C groups, new bone-like tissue was produced in the periphery. However, the degree of cell infiltration remained limited to the periphery.

**FIGURE 8:**
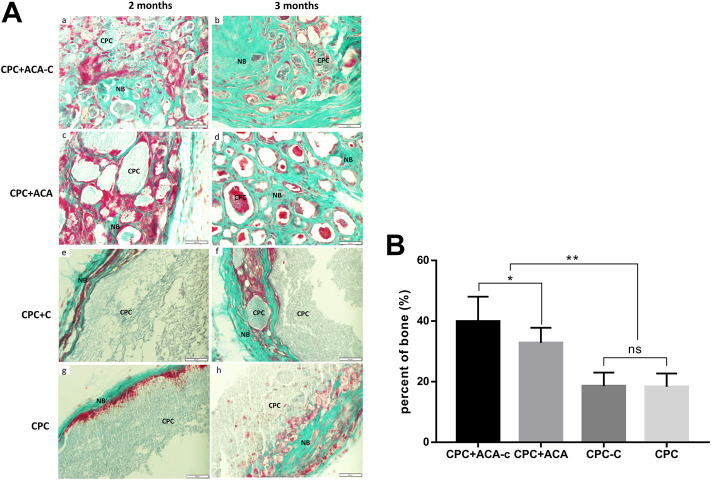
**(A)** Goldner’s trichrome-stained tissue sections of the CPC + ACA-C, CPC + ACA, CPC + C and CPC groups after 2 and 3 months of implantation, bar = 50 μm; **(B)** Newly formed bone areas of Goldner’s trichrome-stained tissue sections after 3 months (NB: newly formed bone; CPC: implanted samples; *: *p <* 0.05, **: *p* < 0.01).

Goldner’s trichrome staining results are presented in Figure 8. In the CPC + ACA and CPC + ACA-C groups, the interior of the scaffold was divided into small sections by nonmineralized osteoid mixed with mineralized bone at 2 months. At 3 months, the osteoid was basically replaced with mineralized bone, and the scaffold degraded into smaller fragments. The interface between mineralized bone and biomaterial comprised incompletely mineralized osteoids. In the CPC and CPC + C groups, a small amount of mineralized bone surrounded the nondegraded scaffold at the periphery of the material at 2 months, and nonmineralized osteoids protruding into the scaffold were observed between the mineralized bone and scaffold. At 3 months, an increased quantity of peripheral mineralized bone with protrusion into the scaffold was evident. Image-Pro Plus was applied to measure the percentage of new bone area of each group at this time point. The data showed that the mineralized areas of the CPC + ACA and CPC + ACA-C groups were significantly greater than those of the CPC and CPC + C groups. Furthermore, the mineralized area of the CPC + ACA-C group was greater than that of the CPC + ACA group.

## Discussion

After combination with hydrogel microcapsules, the injectability and porosity of CPC were significantly increased. The injectability and porosity of a composite mixture with 40% microcapsule content were significantly higher than those of calcium phosphate bone cement, along with good collapse resistance. The compressive strength value was determined to be 3.44 ± 0.77 MPa, which meets the requirements of cancellous bone. With a further increase in the proportion of microcapsules, the composites showed a propensity to collapse and decreased compressive strength. Good injectability (Tariq et al., 2019) and self-setting properties facilitate access of CPC to the bone defect area in a minimally invasive manner, allowing it to solidify *in situ* to adapt to the shape of the cavity. Due to its anti-washout ability, CPC is resistant to loss of bone graft material caused by scouring of body fluid and blood before solidification. Accordingly, we conducted subsequent animal experiments using microcapsule composites with a volume ratio of 40%.

Increasing the porosity of CPC promoted degradation, cell migration, adhesion, proliferation and osteogenic differentiation, along with enhanced angiogenesis and inward bone growth. Moreover, larger pore sizes led to greater cell infiltration and angiogenesis (Klijn et al., 2012; Loh and Choong, 2013). Earlier research (Loh and Choong, 2013; Qiao et al., 2013) suggested that macropores are conducive to cell crawling and vascular growth. Luo et al. (Luo et al., 2021) found that TA scaffolds with a pore size of 400–600 µM had a stronger ability to promote cell adhesion, proliferation and osteogenic differentiation *in vitro*. Several studies have also shown that three-dimensional structures comprising macropores hundreds of microns in size interconnected with pores several microns in size promote cell infiltration and vascular crawling, and the micronanostructures improve the transport of nutrients and metabolic waste and adsorption of bioactive molecules, which contributes to the degradation of scaffold materials and bone formation (Zhang et al., 2013; Guda et al., 2014; Zhou et al., 2019). In experiments, upon addition of microcapsules, the porosity of scaffold materials was significantly increased. The combination with microcapsules at a volume ratio of 40% led to an ∼20% increase in total porosity. Electron microscopy analyses revealed that macropores (hundreds of microns) and pores (tens of microns) were alternately connected with each other. Cell microcapsules not only transport stem cells for scaffold formulations but also form macropores 300–400 µm in size *in situ*. Compared with CPC (with pores a few microns to tens of microns in size), microcapsules promoted the degradation and osteogenesis of biomaterials to a greater extent. A notable limitation of high porosity is a decrease in CPC compressive strength. However, in nonload-bearing areas, such as the craniomaxillofacial region, new bone formation at the expense of mechanical strength is acceptable (Muallah et al., 2021).

In addition, the crosslinking of sodium alginate and Ca^2+^ formed calcium alginate microspheres, and chitosan was used as a coating to electrostatically interact with sodium alginate to form ACA microcapsules, which improved its mechanical strength and stability (Suvarna et al., 2018; Hajifathaliha et al., 2021). Thus, ACA microcapsules can be used as an effective strategy to provide probiotics, proteins and cells to protect against acidity and large amounts of environmental proteases (Cui et al., 2018; Zhang et al., 2020). Cell microcapsules combined with CPC protected cells, reduced the toxicity of CPC solidification, and delivered seed cells to injectable CPC scaffolds. Data from CCK-8, cell live/dead staining and cell adhesion morphology experiments indicate that although cells within ACA microcapsules are protected after CPC solidification, they are still subjected to some damage, resulting in a decline in the cellular state. However, compared with nonencapsulated cells, microcapsule protection enables greater adherence of cells on the surface of CPC after solidification, which subsequently plays a role in the process of osteogenesis after bone cementing. Additionally, microcapsules can degrade into pores *in situ* and release seed cells.

Examination of ectopic osteogenesis on the backs of nude mice revealed stronger osteogenic capability of composite scaffolds with pure microcapsules and BMSC-encapsulating microcapsules. H&E and Masson’s trichrome staining showed that upon addition of microcapsules, cells and blood vessels infiltrated the scaffold. This phenomenon led to fragmentation of the scaffold and degradation of the biomaterial from the inside, prolonged at any time with a gradual decrease in the area of residual material. In the CPC and CPC + C groups, cells and blood vessels were observed only at the periphery, which gradually degraded the scaffold with regeneration of bone, and the biomaterial was degraded from the periphery to the interior. The experimental results suggest that macropores produced by the addition of microcapsules promote the infiltration of cells and blood vessels and accelerate the degradation of scaffolds into bone. Data from Goldner’s trichrome staining were consistent with the H&E results. At 2 months after replantation, osteoids were observed in the CPC + ACA and CPC + ACA-C groups. The material was decomposed into blocks within the scaffold and mixed with mineralized bone. In the CPC and CPC + C groups, only narrow mineralized bone and osteoid wrapped around the scaffold were observed at the periphery, with protrusion of osteoid into the edge of the scaffold to an extent. At 3 months after replantation, the CPC + ACA and CPC + ACA-C groups formed significantly more mineralized bone than the CPC and CPC + C groups. However, compared with the CPC + ACA group, the CPC + ACA-C group showed a stronger osteogenic ability, and the area of mineralized bone was increased from 32.50% ± 6.14%–41.10% ± 8.55%, supporting the theory that BMSCs combined with CPC secrete cytokines or recruit new cells to accelerate scaffold degradation and promote new bone formation. The activities of these cell microcapsules in increasing the pore sizes of scaffolds and accelerating osteogenesis and degradation are valuable for tissue engineering applications.

In bone defects of the nonload-bearing area, CPC + ACA and CPC + ACA-C composite scaffolds could effectively adapt to the shape of the cavity and showed better degradation performance than CPC to match the rate of osteogenesis. Moreover, with the addition of seed cells, these composite biomaterials produced cytokines more rapidly for participation in ectopic osteogenesis.

## Conclusion

In summary, at a 40% volume ratio of cell microcapsules to CPC, composite scaffolds have optimal physicochemical properties that meet the requirements of jaw defect scaffolds, and ACA microcapsules effectively protect cells from toxic effects of the setting reaction of CPC. Ectopic bone formation experiments further confirmed that the combination of CPC with ACA cell microcapsules improves the degradation performance and new bone formation ability, supporting the application prospects of this composite biomaterial in bone tissue engineering.

## Data Availability

The original contributions presented in the study are included in the article/supplementary material, further inquiries can be directed to the corresponding authors.
